# Supplementary material for: Splice-Junction-Based Mapping of Alternative Isoforms in the Human Proteome
Source: Cell Rep. Author manuscript; Available in PMC 2020 Jan 15. (PMC6961840; doi:10.1016/j.celrep.2019.11.026)
Supplement: 2 [file NIHMS1546469-supplement-2.zip › DF1/PXD006675/PulmonaryVein/PulmonaryVein_30_RFWD2_QLEAQLEQIQK.pdf]

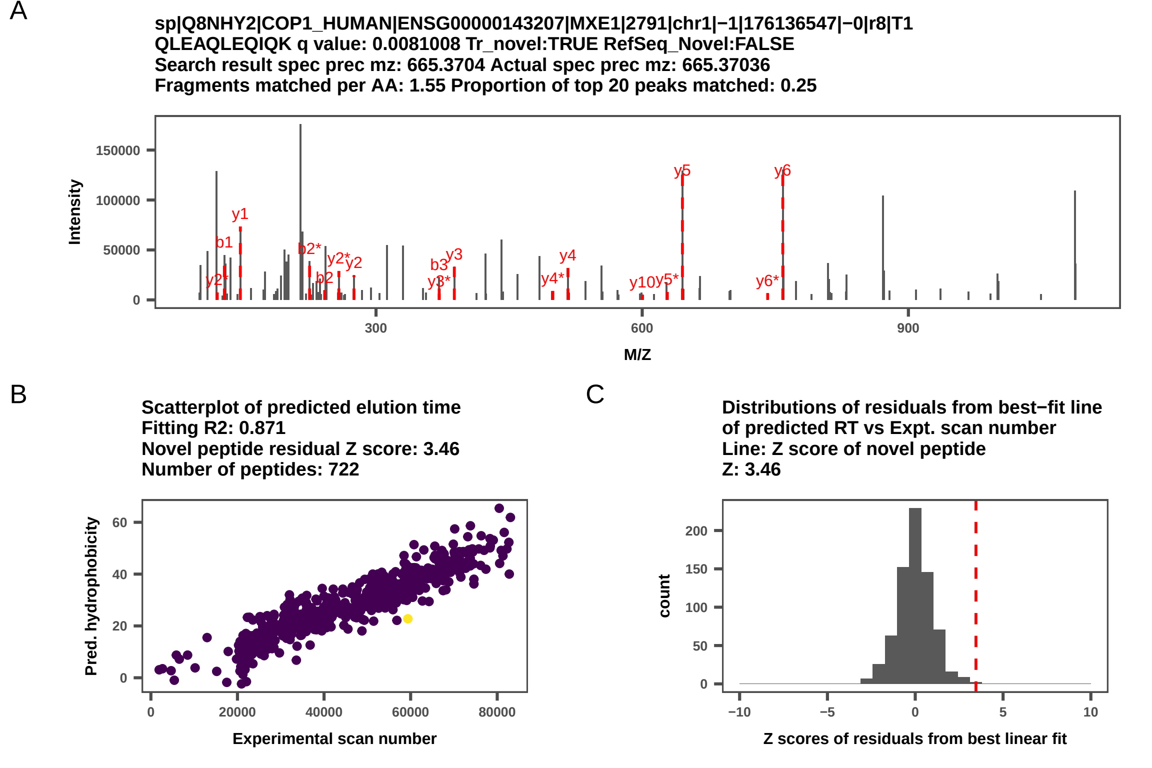
A
sp|Q8NHY2|COP1_HUMAN|ENSG00000143207|MXE1|2791|chr1|−1|176136547|−0|r8|T1
QLEAQLEQIQK q value: 0.0081008 Tr_novel:TRUE RefSeq_Novel:FALSE
Search result spec prec mz: 665.3704 Actual spec prec mz: 665.37036
Fragments matched per AA: 1.55 Proportion of top 20 peaks matched: 0.25
Intensity
150000
100000
50000
0
y1
b1
y2*
b2*
y2*
y2
b2
b3
y3
y3*
y4
y4*
y10
y5*
y5
y6
y6*
300
600
900
M/Z
B
Scatterplot of predicted elution time
Fitting R2: 0.871
Novel peptide residual Z score: 3.46
Number of peptides: 722
Pred. hydrophobicity
60
40
20
0
0
20000
40000
60000
80000
Experimental scan number
C
Distributions of residuals from best−fit line
of predicted RT vs Expt. scan number
Line: Z score of novel peptide
Z: 3.46
count
200
150
100
50
0
−10
−5
0
5
10
Z scores of residuals from best linear fit
